# Surgical Treatment for Unexplained Severe Pain of the Thyroid Gland: Report of Three Cases and Concise Review of the Literature

**DOI:** 10.1155/2011/349756

**Published:** 2011-02-06

**Authors:** Jan van Schaik, Olaf M. Dekkers, Eleonora P. M. van der Kleij-Corssmit, Johannes A. Romijn, Hans Morreau, Cornelis J. H. van de Velde

**Affiliations:** ^1^Department of Surgery, Leiden University Medical Center, P.O. Box 9600, 2300 RC Leiden, The Netherlands; ^2^Department of Endocrinology, Leiden University Medical Center, P.O. Box 9600, 2300 RC Leiden, The Netherlands; ^3^Department of Pathology, Leiden University Medical Center, P.O. Box 9600, 2300 RC Leiden, The Netherlands

## Abstract

Painful thyroid has a limited differential diagnosis. In rare cases, no clear cause can be found after careful clinical, biochemical, and radiological analysis. This may lead to extensive patient morbidity and frustration when symptomatic treatment proves insufficient. Hemithyroidectomy or total thyroidectomy may then be the last resort for doctor and patient. Three cases of unexplained painful thyroid which were successfully treated with hemi or total thyroidectomy are presented. In two cases extensive histological evaluation did not yield a satisfactory explanation for the extreme thyroid pain. In one case histological evaluation of the thyroid revealed Hashimoto's thyroiditis. Review of the literature does not mention surgical treatment for unexplained painful thyroid, and only 15 cases of surgical treatment for painful Hashimoto's thyroiditis are presented. Surgical therapy is a successful final option in the treatment of unexplained painful thyroid and painful Hashimoto's thyroiditis.

## 1. Introduction

Painful thyroid has a limited differential diagnosis. In addition to trauma and nodular hemorrhages, subacute thyroiditis, also known as de Quervain thyroiditis, is the most common cause [1]. Subacute thyroiditis is a mostly bilateral inflammatory disorder causing a slightly swollen, tender thyroid gland, frequently accompanied by thyrotoxicosis. Markedly elevated erythrocyte sedimentation rate and elevated C-reactive protein levels are hallmarks of this disorder. A viral cause has been proposed, as it is often preceded by an upper respiratory tract infection. The condition is self-limiting and symptomatic treatment with analgetics usually suffices. Sometimes corticosteroids are needed. Rare causes of a painful thyroid gland are infection, radiation induced-thyroiditis, malignancy, and neuralgia of the superior laryngeal nerve [1–3]. In case of infection, surgical therapy can be indicated when antibiotic therapy is inadequate or drainage of an abscess is necessary. Nodular hemorrhages and radiation-induced thyroiditis are usually self-limiting conditions [3]. Neuralgia of the superior laryngeal nerve is a complex pain disorder and may require high-dose analgetics and peripheral blockade [2, 4, 5].

In rare cases, no clear cause for a painful thyroid gland can be found after careful clinical, biochemical, and radiological analysis. This may lead to extensive patient morbidity and frustration when symptomatic treatment proves insufficient. Hemithyroidectomy or total thyroidectomy may then be the last resort for doctor and patient. Three cases of unexplained painful thyroid that were successfully treated with surgical therapy are presented, and the differential diagnosis is discussed.

## 2. Case 1

**Figure 1:**
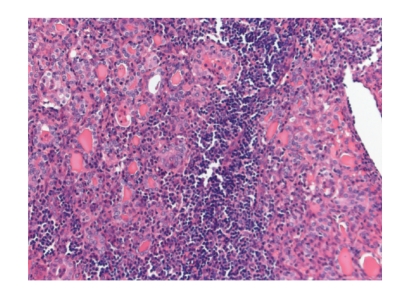
Diffuse hyperplasia with prominent uncomplicated hyperplastic nodes and some lymphocytic thyroiditis (Hematoxylin-eosin, ×200).

A 41-year-old Caucasian woman with a history of a cesarean section and a diagnostic laparoscopy presented with an extremely painful right thyroid gland since four months. Some weeks before the onset of the pain she suffered from an upper respiratory tract infection. There was no fever. The pain was constant and increased on swallowing. The patient was initially treated by the family physician. Analgetics such as nonsteroidal anti-inflammatory drugs and morphinomimetics did not decrease the pain. On physical examination the right thyroid lobe was not enlarged but extremely painful on palpation. There were no obvious thyroid nodes. The left thyroid lobe was normal. There were no enlarged lymph nodes in the neck. Laboratory tests showed euthyroidism: thyroid stimulating hormone (TSH) level was 0.59 mU/L (normal value 0.4–4.4 mU/L), free thyroxine (T4) level was 19.4 pmol/L (normal value 10–24 pmol/L), and triiodothyronine (T3) level was 1.7 nmol/L (normal value 1.3–2.6 nmol/L). Thyroid peroxidase (TPO) antibody level was low (<10 ku/L). Leukocyte count (6,400/mm^3^), erythrocyte sedimentation rate (11 mm/h), and C-reactive protein level (7 mg/dL) were within normal limits. Ultrasonography showed multiple nodes in the right thyroid lobe with a maximum diameter of 12 mm without any signs of haemorrhage or abscess. The dental surgeon and otolaryngologist excluded referred pain from dental or nasopharyngeal pathology. The patient was treated with analgetics and subsequently corticosteroids for several weeks based on the presumed diagnosis of subacute thyroiditis. However, this did not resolve the symptoms. The pain was increasingly invalidating and led to absence of work. Finally, 6 months after onset of symptoms, a right hemithyroidectomy was performed. The specimen measured 5 × 3 × 3 cm. Histological study revealed diffuse hyperplasia with prominent uncomplicated hyperplastic nodes and some mild lymphocytic thyroiditis (Figure 1). Immediately after surgery the pain had disappeared completely. Up to one year following surgery the patient was free of pain and had a normal thyroid function.

## 3. Case 2

**Figure 2:**
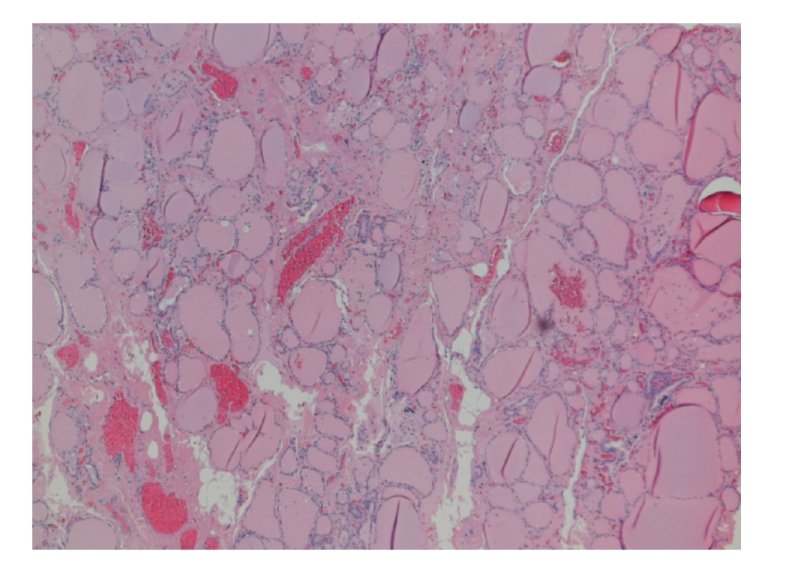
Nodular hyperplasia with degenerative changes, a limited lymphocytic thyroiditis and adipose metaplasia of thyroid stroma. Some signs of bleeding and previous bleeding are seen (Hematoxylin-eosin, ×40).

A 41-year-old Caucasian woman with a history of laparoscopic gastric banding for morbid obesity and a laparoscopic cholecystectomy presented with an extremely painful right thyroid lobe. Since several months she had experienced palpitations and heat intolerance, and since three weeks extreme pain had developed in the right thyroid lobe during a period of fever and malaise. She had not noticed any enlargement of the thyroid gland. There had been no weight loss or other physical changes. Analgesic therapy did not relieve the symptoms. On physical examination there were no signs of thyrotoxicosis. There was a slightly enlarged left thyroid lobe without any palpable nodes and a palpable node of approximately 2 cm in the right lobe, which was very painful on examination. There were no enlarged lymph nodes in the neck. Laboratory tests revealed thyrotoxicosis: TSH level was 0.02 mU/L (normal value 0.4–4.4 mU/L) and free thyroxine (T4) level 60.7 pmol/L (normal value 10–24 pmol/L). There were no detectable thyroid-stimulating antibodies or TPO antibodies. There was no leukocytosis (4,400/mm^3^), erythrocyte sedimentation rate was 29 mm/h, and there was a slight increase in C-reactive protein (23 mg/dL). A Tc-99M scan showed a slightly enlarged thyroid with normal 24-hour uptake, and a cold node on the right side of 4 cm, as well as a node on the left side (3 cm) with normal uptake. Fine-needle aspiration yielded no conclusive diagnosis. The otolaryngologist excluded any pharyngeal or tracheal pathology. The following causes were considered in the differential diagnosis: subacute thyroiditis of de Quervain or an early hyperthyroid phase of Hashimoto's thyroiditis (Hashitoxicosis). The thyroid function returned to normal within two months without any antithyroid therapy. However, the right hemithyroid remained extremely painful. The patient was treated with nonsteroidal anti-inflammatory drugs and morphinomimetics for several weeks without any improvement of symptoms. Finally, four months after onset of pain, a right hemithyroidectomy was performed. The specimen measured 4 × 4 × 2 cm. Histological study showed some nodular hyperplasia with degenerative changes, a limited lymphocytic thyroiditis, and a focal area with sclerosis. Elsewhere, bleeding and signs of previous bleeding were seen. Remarkable was the presence of adipose metaplasia of thyroid stroma (Figure 2). Immediately after surgery the pain had disappeared completely. Up to several months after surgery, the patient was free of pain and had a normal thyroid function.

## 4. Case 3

**Figure 3:**
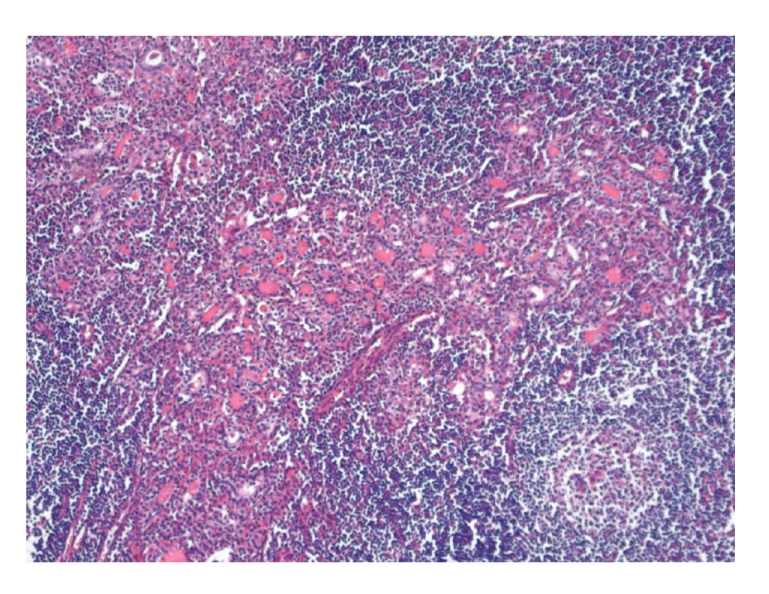
Severe lymphocytic infiltration with formation of lymphatic follicles and Hürthle cells (Hematoxylin-eosin, ×100).

A 17-year-old Caucasian woman with no prior medical history presented with an extremely painful thyroid. She had noticed gradual enlargement of the thyroid gland since five years, but more rapid growth during the last six months, accompanied by pain on the right side. Moreover, she had difficulty swallowing because of pain, resulting in decreased food intake and 10 kg weight loss during the last six months. In addition, she complained of a coarse voice since several months. There was no fever. Analgetic therapy did not relieve the symptoms. On physical examination there were no signs of hypo- or hyperthyroid function. There was a WHO grade II goiter without palpable nodules which was extremely painful on examination. There were no enlarged lymph nodes in the neck, and there were no signs of respiratory distress. Laboratory tests showed biochemical euthyroidism: TSH level was 3.77 mU/L (normal value 0.4–4.4 mU/L), free thyroxine (T4) level was 13.4 pmol/L (normal value 10–24 pmol/L), and triiodothyronine (T3) level was 2.1 nmol/L (normal value 1.3–2.6 nmol/L). TPO antibody level was increased (459 ku/L). There was no increased leukocyte count (3,400/mm^3^) or increased erythrocyte sedimentation rate (5 mm/h). An I-123 scan showed a homogenously enlarged thyroid (slightly more increased on the right side) with a 24-hour uptake of 42%. The otolaryngologist excluded any pharyngeal or tracheal pathology. Esophagogastroscopy revealed no pathology explaining difficulty swallowing or weight loss. Fine-needle aspiration of the thyroid revealed lymphocytic thyroiditis, in accordance with the diagnosis of Hashimoto's thyroiditis. The patient was treated with nonsteroidal anti-inflammatory drugs and corticosteroids for several months without any improvement of symptoms. Finally, eight months following onset of pain, a total thyroidectomy was conducted. Histological study indeed showed a very severe lymphocytic thyroiditis with formation of lymphatic follicles (Figure 3). Immediately after surgery the pain had disappeared completely. Up to several years following surgery the patient was free of pain.

## 5. Discussion

This case series describes three patients with extreme, invalidating pain located within the thyroid gland, which could not adequately be managed by symptomatic treatment. The complaints resolved completely and immediately upon surgical removal of the painful thyroid tissue.

The clinical presentation in the first case is reminiscent of subacute thyroiditis which is sometimes preceded by a viral upper respiratory tract infection. However, unilateral pain, normal levels of C-reactive protein, and a normal erythrocyte sedimentation rate are not in agreement with this diagnosis. Moreover, subacute thyroiditis is often characterized by thyrotoxicosis followed by hypothyroidism. The symptoms usually improve dramatically following the administration of nonsteroidal anti-inflammatory drugs. Finally, the histological specimens did not show the typical changes expected in subacute thyroiditis nor yielded any other cause for the patient's complaints.

In the second case, the differential diagnosis included early hyperthyroid phase of Hashimoto's thyroiditis (HT) and subacute thyroiditis. Unilateral pain, however, is not typical for either condition. The absence of thyroid antibodies would support subacute thyroiditis. However, a normal 24-hour I-123 uptake or Tc-99M scan and the only slightly elevated erythrocyte sedimentation rate and C-reactive protein levels are more in favour of HT. We could not document histological changes confirming HT nor subacute thyroiditis.

Histological evaluation of the thyroid gland of the third case clearly identified HT. This autoimmune disease is characterized by painless goiter, and hypothyroidism is sometimes preceded by a hyperthyroid phase [1]. TPO antibodies are usually elevated, but they may be low or even absent in HT. Rarely, it may present as a painful goiter and sometimes clinically resembles subacute thyroiditis. Although painful HT often presents with fever, transient thyrotoxicosis, elevated erythrocyte sedimentation rate, and elevated levels of C-reactive protein, patients may also be euthyroid or hypothyroid, and infection parameters may be normal [10]. The diagnosis of HT in the case of an extremely painful thyroid is therefore typically based on histological studies. Although painful HT has been previously described, the literature is limited to case series [6–12]. Initial treatment for painful HT is typically symptomatic with nonsteroidal anti-inflammatory drugs and corticosteroids or may be focused on hormone replacement therapy. Quite often this might not be sufficient. In these cases surgical treatment has proven a successful option, in accordance with our observation [6–9]. Clinical features and pain relief following surgery of all patients presented in the literature that were surgically treated for painful HT are summarized in Tables 1 and 2. 

Speculation on the cause of pain in the first two cases includes atypical viral infection since both patients had experienced a prodromal period of fever and malaise. Another speculation is that even uncomplicated thyroid nodes might cause severe pain in exceptional cases.

The differential diagnosis of unilateral thyroid pain includes superior laryngeal neuralgia, a complex pain disorder which is thought to represent a “referred pain” from the pharyngeal and laryngeal regions. A viral infection has also been proposed. A substrate for superior laryngeal neuralgia is seldom found. The pain is typically located between the thyroid and the ear and is described as paroxysmal and “electrical”. There is usually a trigger point, and episodes may be accompanied by nausea or excessive lacrimation. Treatment is symptomatic and may require high-dose analgetics and peripheral blockade [2, 4, 5]. However, none of the above-mentioned clinical presentations resembled superior laryngeal neuralgia. 

In conclusion, we describe three patients with extreme invalidating pain which severely interfered with daily activities and work. In all cases symptomatic treatment was not successful. The pain was immediately relieved by hemithyroidectomy or total thyroidectomy. Remarkably, extensive histological evaluation did not yield a satisfactory explanation for the extreme thyroid pain in two cases, and in one case, HT was found. This indicates that surgical therapy is a successful final option in the treatment of unexplained painful thyroid and painful HT.

## Figures and Tables

**Table 1:** Characteristics of fifteen patients with painful Hashimoto's thyroiditis who underwent thyroidectomy.

Authors	No.	Age/sex	Symptoms	ESR (mm/h)	CRP (mg/dL)	TT4 (*μ*g/dL)	FT4 (ng/dL)	TT3 (ng/dL)	TSH (mU/L)	24-h RAIU (%)	MCHA/ TGHA Titer	FNA
Normal range				0–20	<0.3	5–12	0.8–2.0	120–190	0.5–6.0	10–35%	0/0	
Kon and DeGroot [6]	1	36/F	Daily neck pain and swelling, 5 months	11	1.3	8.3	NM	NM	1.5	23%	320/160	ND
	2	26/F	HT/hypothyroid on L-T4, painful goiter, 1-2 years	20	ND	6.9	NM	NM	9.2 (L-T4 175 *μ*g)	27%	20,480/40	HT
	3	39/F	HT/hypothyroid on L-T4, episodic throat pain radiating to chin and ears, 3 years	10	<0.3	12.1	NM	NM	1.46 (L-T4 112 *μ*g)	ND	6,400/ND	Inconclusive
	4	48/F	Initially “like SAT”, episodic neck pain, 7 months	11–39 (NR 1–39)	<0.3	5.9	NM	NM	4.6	Initial 2%, after 7 months 28%	0/0	Florid lymphocytic thyroiditis
	5	28/F	HT, painful goiter radiating to ears, odynophagia, 1 month	11–17	ND	5.0	NM	432	4	25%	>20,480/10,240	HT, giant cells
	6	22/F	L-T4 for diffuse goiter, pain right thyroid lobe to right ear, dysphagia, 2 months	18	ND	10.8	NM	NM	0.3 (L-T4 100 *μ*g)	5h 7%, 31 h 17%	1,280/40	ND
	7	41/F	Constant painful goiter on L-T4, especially right side, odynophagia, hoarseness, 5 months	16–22	1.2 (NR 0–1.8)	11	NM	NM	0.8 (L-T4 175 *μ*g)	ND	0/0	HT, giant cells

Ohye et al. [7]	1	24/M	Fever, neck pain	0.6	3.1	NM	1.22	NM	7.72	ND	409,600/25,600	HT
	2	65/F	Neck pain	51	ND	NM	1.22	NM	ND	0.6%	102,400/409,600	ND
	3	56/F	Fever, neck pain, swelling	66	ND	NM	0.72	NM	8.75	31.9%	400/1,638,400	HT
	4	62/F	Fever, neck pain	ND	9.0	NM	1.61	NM	0.063	ND	3.3 U/mL^a^, 13,300 U/mL^b^	ND
Gourgiotis et al. [8]	1	56/F	Prior left hemithyroidectomy for Graves' disease and HT, episodic neck pain, hoarseness, 4 years	19	NM	NM	1.80	121	0.05 (L-T4 150 *μ*g)	ND	611 U/mL^a^, 112 U/mL^b^	Hürthle cells, no thyroiditis
	2	32/M	Neck pain, swelling, dysphagia, HT, 2 years	5	NM	NM	1.70	320	0.60 (L-T4 175 *μ*g)	ND	320/100	ND

Zimmerman et al. [9]	1	52/F	Episodic neck pain, sore throat, 5 years	7	NM	5.4	NM	NM	NM	35%	6,400/0	Lymphocytic thyroiditis
	2	41/F	Tender goiter, 6 months	16	NM	4.8	NM	NM	NM	23%	ND	Lymphocytic thyroiditis

ND: not determined; NM: not mentioned; NR: normal range; MCHA: antimicrosomal hemagglutination; TGHA: antithyroglobulin hemagglutination; HT: Hashimoto's thyroiditis; ^a^anti-TPO antibody (normal range, <0.3 U/mL); ^b^antithyroglobulin antibody (normal range, <0.3 U/mL); L-T_4_: L-thyroxine.

**Table 2:** Pain relief to treatment and surgical pathology in fifteen cases of painful Hashimoto's thyroiditis.

Authors	No.	Pain relief to T4	Pain relief to steroids	Pain relief to aspirin or NSAIDS	Time from pain onset to surgery (years)	Pain relief after thyroidectomy	Type of surgery	Pathology
Kon and DeGroot [6]	1	No	No	Not given	2	Total, permanent	NTT	Focal lymphocytic thyroiditis
	2	No	No	Not given	2	Total, but recurrence after 1 year	NTT	Hashimoto's thyroiditis, incidental pappilary cancer
	3	No	Rapid but relapsing	Not given	3.1	Total, permanent	NTT	Near end stage thyroiditis
	4	No	Rapid but relapsing	No to NSAID	1.1	Total, permanent	STT	Focal lymphoid infiltrates, sparse Hürthle cells
	5	Partial	Not given	Unable to tolerate NSAID	6.2	Total, permanent	STT	Fibrosis, large reactive lymphocyte follicles, Hürthle cell clusters
	6	No	Not given	No	0.5	Partial	STT	Diffuse lymphocytic thyroiditis
	7	No	Rapid but relapsing	Partial	0.6	Total, relapsed	STT	Hashimoto's thyroiditis

Ohye et al. [7]	1	Yes but relapsing	Rapid but relapsing	NM	2.7	Total, permanent	TT	Severe fibrosis, lymphocytic infiltration
	2	No	Rapid but relapsing	NM	4.0	Total, permanent	TT	Severe fibrosis, lymphocytic infiltration
	3	No	Rapid but relapsing	NM	2.0	Total, permanent	TT	Severe fibrosis, lymphocytic infiltration
	4	Not given	Rapid but relapsing	NM	0.75	Total, permanent	TT	Giant cells, mild fibrosis, remaining follicular structure

Gourgiotis et al. [8]	1	No	Yes, relapsing	NM	>10	Total, permanent	TT	Severe fibrosis, lymphocytic infiltration, Hürthle cells
	2	No	NM	NM	2.1	Total, permanent	TT	Lymphocytic thyroiditis, focal Hürthle cells

Zimmerman et al. [9]	1	No	No	Yes, relapsing	>5	Total, permanent	TT	Lymphocytic infiltration
	2	No	Yes, relapsing	Not given	0.5	Total, permanent	TT	Lymphocytic thyroiditis

NTT: near total thyroidectomy; STT: subtotal thyroidectomy; TT: total thyroidectomy.
